# TF-STNet: A Time–Frequency Dual-Branch Spatiotemporal Network for NWP-to-Station Bias Correction

**DOI:** 10.3390/e28091004

**Published:** 2026-09-08

**Authors:** Zhao Wang, Shuai Chen, Yunbo Yang, Bo Wang, Bihe Xu, Yangliao Geng

**Affiliations:** 1 State Key Laboratory of Renewable Energy Grid-Integration, China Electric Power Research Institute, Beijing 100192, China; wangzhao@epri.sgcc.com.cn (Z.W.); chenshuai@epri.sgcc.com.cn (S.C.); yangyunbo@epri.sgcc.com.cn (Y.Y.); wangbo@epri.sgcc.com.cn (B.W.); 2 Key Laboratory of Big Data & Artificial Intelligence in Transportation (Ministry of Education), Beijing Jiaotong University, Beijing 100044, China; xbhnew@bjtu.edu.cn

**Keywords:** numerical weather prediction, spatiotemporal learning, time–frequency representation, graph neural networks, station-scale forecasting, meteorological post-processing, spectral attention

## Abstract

Accurate station-scale weather forecasts support renewable-energy and transportation operations, yet gridded numerical weather prediction (NWP) is affected by representativeness errors and regional biases when transferred to irregularly distributed stations. We propose TF-STNet, a time–frequency dual-branch spatiotemporal network for NWP-to-station bias correction. A station-centered K-nearest-neighbor (KNN) operator retains multiple local NWP trajectories. The time-domain pathway separately encodes recent observations and future NWP, aligns them over the forecast horizon using gated dilated causal convolutions, and propagates lead-resolved states through a coordinate-conditioned directed station graph. The frequency-domain pathway learns spectral weights and applies separate attention to amplitude and phase across neighboring NWP cells. Prediction-level fusion combines the two station forecasts by variable, station, and lead time. The evaluation uses hourly data for wind speed, pressure, relative humidity, and temperature from 455 stations in Hebei, Shandong, Fujian, and Sichuan. Across five independent runs on 16 region–variable tasks, TF-STNet achieves the lowest mean absolute error (MAE) in 15 tasks and the highest Pearson correlation coefficient (PCC) in 15 tasks; its pressure MAE reduction relative to the strongest learned comparator ranges from 16.2% to 57.4% across the four regions. It has lower MAE than raw NWP in seven of eight high-wind or rapid-change event tests and than simple pressure model-output-statistics corrections in all four regions. The Shandong–temperature task and the Hebei–high-wind case illustrate the limits of the present point-forecast formulation.

## 1. Introduction

Accurate station-scale forecasting is a fundamental task in meteorology, supporting weather monitoring, early warning, environmental management, and a wide range of weather-sensitive activities; recent limited-area AI models further underscore the demand for high-resolution forecasts of surface meteorological variables [1]. Modern observation networks and numerical weather prediction (NWP) systems generate large, heterogeneous spatiotemporal datasets, creating substantial opportunities for artificial intelligence and neural networks to learn nonlinear relationships across variables, locations, and forecast lead times, as demonstrated by large-scale Earth-system foundation models [2]. NWP remains indispensable because it provides physically consistent guidance throughout the forecast horizon. However, its grid cells represent area-averaged atmospheric states, whereas station observations describe specific locations. Differences in elevation, land cover, coastal exposure, and unresolved local processes consequently produce systematic and time-varying discrepancies between gridded forecasts and station measurements. Conventional spatial interpolation can reduce this mismatch in spatial support, but it cannot learn recurring grid-to-station relationships or jointly represent their temporal evolution and spatial dependence; recent neural downscaling studies demonstrate the value of learned mappings for reconstructing fine-scale meteorological structure from coarse fields [3].

Statistical post-processing provides a direct way to estimate such forecast–observation relationships from historical data. Classical model output statistics and ensemble post-processing established this principle, while neural post-processing extended it to nonlinear and multivariate mappings for both deterministic and probabilistic products [4,5]. Recent work has also formulated coarse-to-fine rainfall downscaling as conditional distribution learning with spatial dependence [6]. Nevertheless, performance remains sensitive to the forecasting method and to changes in nonlinearity, dependence, and temporal complexity [7]. These challenges are especially pronounced in regional meteorology: wind speed, pressure, relative humidity, and temperature differ in scale, persistence, periodicity, and terrain sensitivity, and their NWP errors vary across coastal, plain, plateau, and basin environments. An effective correction model must therefore accommodate multiple forms of heterogeneity without allowing one variable or information source to dominate the learned representation.

Meanwhile, data-driven global weather forecasting has advanced rapidly. FourCastNet introduced adaptive Fourier neural operators for high-resolution global prediction [8]; Pangu-Weather, GraphCast, and FuXi demonstrated skillful medium-range forecasting through three-dimensional neural networks, graph-based message passing, and cascaded lead-time models [9,10,11]; and generative systems subsequently extended these advances to probabilistic forecasting [12]. Hybrid neural general circulation models combine learned components with differentiable physical solvers [13], while WeatherBench 2 provides a standardized framework for comparing global systems [14]. These models offer broad skill and rapid inference, but they reconstruct atmospheric states on regular grids and are optimized for large-scale performance rather than the terrain, exposure, and measurement characteristics of individual stations. ASTAFN directly addresses this gap by using proxy-station learning to align weather foundation model outputs with station observations and adaptively fuse their spatiotemporal features [15]. More generally, model rankings vary with the target variable, lead time, and verification regime [16]. Thus, advances in global forecasting strengthen the physically grounded guidance available for local prediction but do not eliminate the need for dedicated station-level correction.

At the station scale, NWP correction has a distinctive spatiotemporal structure. Historical observations describe the current station state and its recent local discrepancy, whereas NWP is the only input that spans the future forecast horizon. The two sources are therefore temporally adjacent but statistically asymmetric: one is measured and retrospective, while the other is model-generated and prospective. In addition, weather systems couple irregularly distributed stations, and these dependencies may be directional or shaped by factors beyond geographic distance. Graph WaveNet learns latent adjacency jointly with temporal dynamics [17], MegaCRN represents recurring spatial patterns through meta-graphs [18], and MTGNN infers multivariate relations from data [19]. Gated temporal representations can also benefit from learned graph structure when spatial dependencies evolve [20]. However, these graph forecasters are generally formulated for node-aligned temporal inputs and do not explicitly reconcile measured history with gridded future guidance.

NWP correction residuals also exhibit complementary structure in the time and frequency domains. Temporal order and short-range context are essential to representing fronts, ramps, and abrupt local transitions, whereas recurrent or slowly varying errors across the full forecast horizon are often more directly expressed through spectral content. Attention-based forecasters capture long-range dependence [21,22]; PatchTST learns patch-wise, channel-independent representations [23]; DLinear demonstrates the strength of decomposed linear structure [24]; and Temporal Fusion Transformers incorporate heterogeneous covariates in multi-horizon forecasting [25]. Frequency-aware models such as Autoformer, FEDformer, TimesNet, and FreTS further demonstrate the value of periodic and multi-scale representations [26,27,28,29], while PHAT explicitly models heterogeneous and dynamically changing periods across variables [30]. More broadly, time–frequency analysis can reveal signal organization that is difficult to isolate in either domain alone [31]. This distinction is particularly relevant to bias correction: an NWP trajectory may reproduce a diurnal cycle but misestimate its amplitude, or it may capture the strength of a change while shifting its arrival time. Although both cases produce pointwise errors, they require different forms of correction.

Taken together, NWP-to-station correction requires resolving three coupled mismatches: measured history must be aligned with model-generated future guidance, regular NWP grids must be translated to irregular station locations, and lead-specific temporal changes must be distinguished from horizon-scale spectral errors. Existing graph and frequency-aware forecasters address individual aspects of this problem but generally assume that their inputs already share common temporal and spatial support. They also do not explicitly distinguish whether neighboring NWP trajectories are reliable in oscillation amplitude, timing, or both. Naively fusing all inputs at an early stage would therefore force heterogeneous temporal axes, spatial structures, and feature semantics into one latent representation, potentially weakening source-specific learning and obscuring abrupt lead-dependent changes. These considerations motivate an architecture that preserves source- and domain-specific representations until they can be combined at a common station-forecast level.

To address these limitations, this paper introduces TF-STNet, a time–frequency dual-branch spatiotemporal network that preserves source and representation boundaries until meaningful alignment is possible. A shared station-centered KNN operator retains multiple local NWP trajectories instead of collapsing them into a single interpolated value. The time-domain pathway separately encodes observation history and future NWP, projects the historical state onto the forecast horizon, and propagates the fused lead-resolved features through a coordinate-conditioned directed station graph. In parallel, the frequency-domain pathway learns the relevance of spectral components and applies separate amplitude and phase attention across neighboring NWP cells, allowing the preferred spatial evidence to vary by station and oscillatory scale. Each pathway first produces a station-level forecast; prediction-level fusion then combines outputs with identical physical dimensions and adjusts their contributions by variable, station, and lead time. The resulting pathways act as complementary correction estimators: the time-domain pathway emphasizes causal local evolution and inter-station propagation, whereas the frequency-domain pathway targets horizon-scale distortions in oscillatory strength and timing.

The main contributions are as follows:

1. We propose TF-STNet, a time–frequency fusion framework that jointly captures lead-specific local departures and horizon-scale distortions in the amplitude and timing of NWP variations. By combining complementary station-level estimates only after each representation has specialized, the framework avoids premature feature mixing and adapts its correction to each variable, station, and forecast lead.
2. TF-STNet introduces a station-centered amplitude–phase attention mechanism that processes Fourier amplitude and phase separately across neighboring NWP cells. This mechanism distinguishes errors in oscillatory strength from timing shifts and selects the most informative local evidence for each component.
3. Experiments across 455 stations, four meteorological variables, and four geographically distinct Chinese regions yield 16 region–variable tasks. Across five independent runs, TF-STNet achieves the lowest mean MAE and the highest mean PCC in 15 of the 16 tasks; Shandong–temperature is the only task for which another method has a lower mean MAE.

## 2. Problem Formulation and Proposed Method

### 2.1. Problem Definition

**Figure 1:**
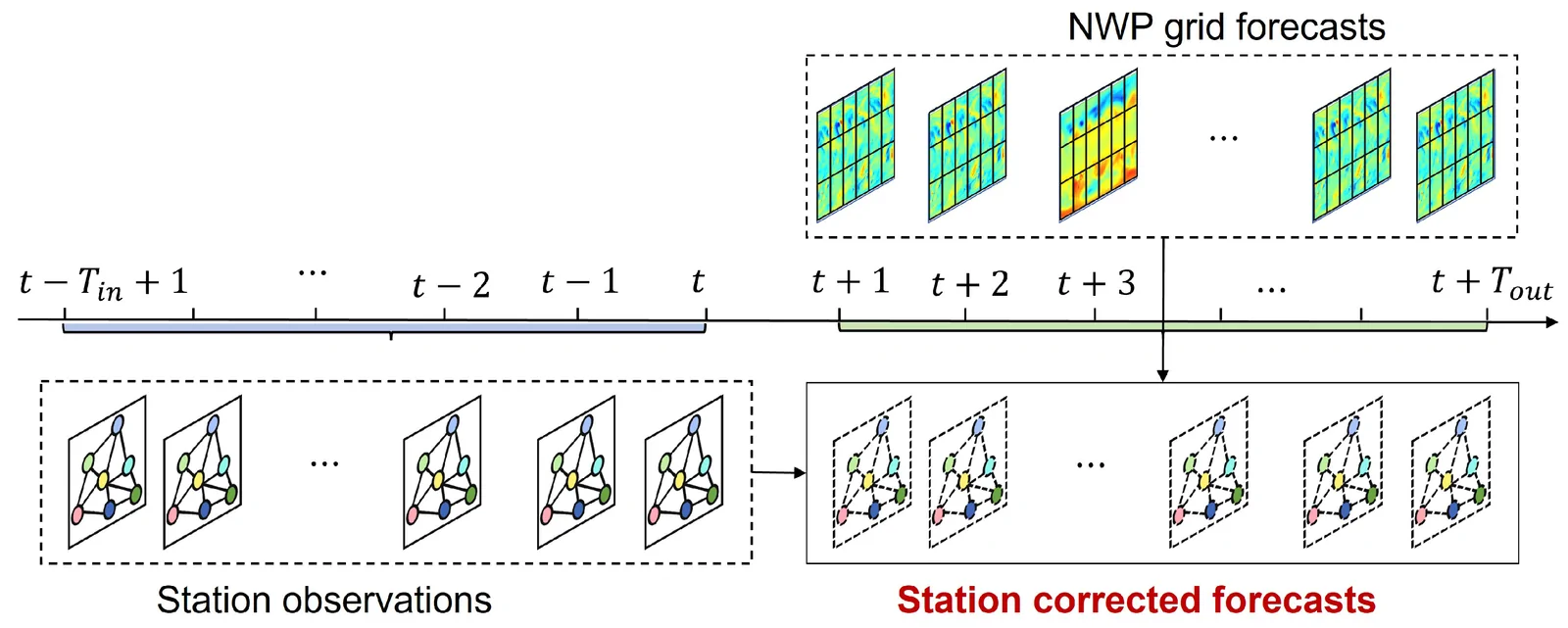
Input–output definition of station-level NWP bias correction. Historical station observations end at the forecast origin, while gridded NWP provides guidance over the future horizon. The target is the corresponding corrected trajectory at each station. The different colors in the NWP grid (top right) represent the magnitude of the target weather variable (such as temperature), with warmer colors indicating higher values.

Let a forecast be issued at time *t* for *N* target stations and *C* meteorological variables. The available inputs are a recent sequence of station observations and a gridded NWP trajectory over the future forecast horizon; the target is the corresponding future station sequence. Throughout this section, bold symbols denote vectors, matrices, or higher-order tensors; subscripts index tensor axes, while descriptive superscripts label representations rather than powers. Omitting the batch dimension, the principal tensors are

(1) $$X\in R^{T_{\mathrm{in}}\times N\times C},\;E\in R^{T_{\mathrm{out}}\times N_{\mathrm{lat}}\times N_{\mathrm{lon}}\times C},\;Y\in R^{T_{\mathrm{out}}\times N\times C},$$

where $T_{\mathrm{in}}$ and $T_{\mathrm{out}}$ are the numbers of hourly input and output steps, respectively; both equal 24 in this study. The number of variables is C=4, corresponding to wind speed, pressure, relative humidity, and temperature, while $N_{\mathrm{lat}}$ and $N_{\mathrm{lon}}$ denote the numbers of NWP grid points along the latitude and longitude axes. Station and NWP-grid coordinates are denoted by $S\in R^{N\times 2}$ and $G\in R^{N_{\mathrm{lat}}\times N_{\mathrm{lon}}\times 2}$, respectively. The correction model learns the mapping

(2) $$\hat{Y}=f_{\theta }\left(X,E,S,G\right),$$

with learnable parameters θ. The observation sequence X ends at the forecast origin, whereas E covers valid times t+1 to $t+T_{\mathrm{out}}$; no future station measurement is available to the model. As illustrated in Figure 1, the task is therefore to condition a physically generated future NWP trajectory on recent station behavior and spatial context, rather than to extrapolate the future from observations alone.

### 2.2. TF-STNet Overview and Station-Centered NWP Extraction

**Figure 2:**
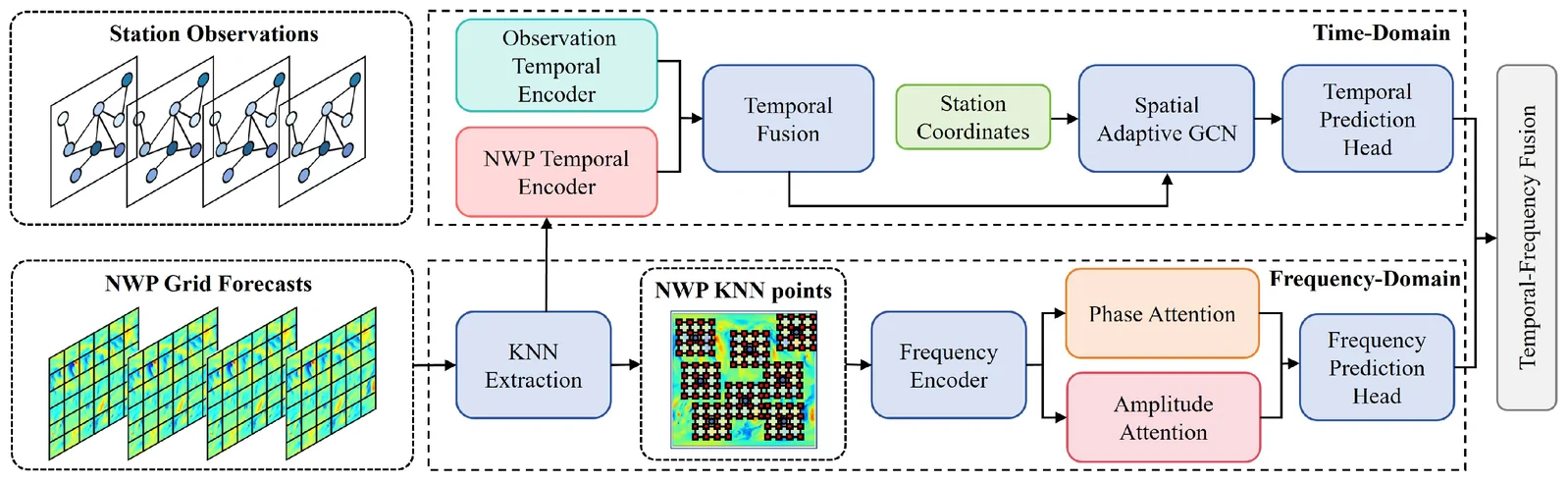
Architecture of TF-STNet. Shared KNN extraction supplies the same local NWP trajectories to two complementary pathways. Source-aware temporal alignment and adaptive graph propagation produce the time-domain candidate, while spectral weighting and amplitude–phase attention produce the frequency-domain candidate. Prediction-level fusion combines the two station-level trajectories. The different colors in the NWP grid (bottom left) represent the magnitude of the target weather variable (such as temperature), with warmer colors indicating higher values.

TF-STNet addresses the source, spatial-support, and representation mismatches identified in the Introduction through four stages (Figure 2): station-centered NWP extraction, parallel time- and frequency-domain pathways, and prediction-level fusion. The extraction stage maps the regular NWP grid to a common local support around each irregular station. The time-domain pathway learns lead-specific corrections from recent observations, future NWP, and inter-station dependence, whereas the frequency-domain pathway targets horizon-scale distortions in oscillatory strength and timing. Their outputs are fused only after both pathways have produced station-level trajectories. The hidden dimension is denoted by *D* and the number of retained NWP cells per station by *K*, and superscripts *t* and *f* identify time- and frequency-domain quantities. Subscripts attached to MLP and Head distinguish independently parameterized modules by their roles.

The shared extraction stage converts the regional NWP lattice into a compact station-centered representation. For station i∈{1,…,N} with coordinate vector $s_{i}$, let $N_{K}\left(i,k\right)=\left(a_{ik},b_{ik}\right)$ denote the latitude–longitude index pair of its *k*th-nearest NWP grid point, where k∈{1,…,K}, $a_{ik}\in \left\{1,\ldots ,N_{\mathrm{lat}}\right\}$ and $b_{ik}\in \left\{1,\ldots ,N_{\mathrm{lon}}\right\}$. The local NWP tensor is

(3) $$E^{K}=\mathrm{KNN}\left(E,S,G\right)\in R^{T_{\mathrm{out}}\times N\times K\times C},\;E_{\tau ,i,k,c}^{K}=E_{\tau ,a_{ik},b_{ik},c},$$

where $\tau \in \{1,\ldots ,T_{\mathrm{out}}\}$ indexes forecast lead and c∈{1,…,C} indexes meteorological variable. Rather than committing to a single interpolated grid value, Equation (3) retains *K* surrounding NWP trajectories as a physically meaningful candidate set. Subsequent learned operations determine how these candidates contribute to each correction. Sharing $E^{K}$ across both pathways ensures that their differences arise from time- and frequency-domain representations rather than unequal spatial inputs.

From this shared local context, the time-domain pathway produces a lead-resolved candidate ${\hat{Y}}^{t}$ after source-aware temporal alignment and adaptive graph propagation. In parallel, the frequency-domain pathway learns spectral relevance, evaluates amplitude and phase evidence across neighboring cells, and produces ${\hat{Y}}^{f}$. Because both candidates use the same station, variable, and lead-time coordinates, they can be combined directly without forcing their intermediate features into an artificial common latent space.

### 2.3. Time-Domain Pathway

#### 2.3.1. Source-Aware Temporal Encoding

Historical observations and future NWP are processed by separate temporal encoders because they differ in temporal direction, spatial organization, and error characteristics. Both encoders use gated dilated causal convolutions, but they do not share parameters: X is a measured history ending at *t*, whereas $E^{K}$ is a model-generated future sequence with an additional local-neighbor axis. For a generic hidden sequence U, a dilated causal convolution with kernel Ψ at temporal index τ is

(4) $${\left(U{*}_{\delta }\Psi \right)}_{\tau }=\sum_{r=0}^{L_{c}-1}U_{\tau -\delta r}\Psi_{r},$$

where $L_{c}$ is the kernel length, δ is the dilation factor, *r* indexes the kernel tap, and unavailable indices are replaced by left causal padding. Increasing δ across successive blocks expands the receptive field without discarding the temporal order.

Each block applies two convolutions to form a candidate state and a data-dependent gate:

(5) $$T_{\delta }\left(U\right)=\tanh\left(U{*}_{\delta }\Theta_{a}+b_{a}\right)⊙\sigma \left(U{*}_{\delta }\Theta_{g}+b_{g}\right),$$

where $\Theta_{a}$ and $\Theta_{g}$ are the learnable convolution kernels for the candidate and gate, $b_{a}$ and $b_{g}$ are their biases, ⊙ denotes element-wise multiplication, and σ(·) is the sigmoid function. Residual connections preserve short-range fluctuations that could otherwise be attenuated by the dilated stack.

The observation encoder produces $H^{\mathrm{obs}}\in R^{T_{\mathrm{in}}\times N\times D}$, while the NWP encoder produces $H^{\mathrm{nwp}}\in R^{T_{\mathrm{out}}\times N\times K\times D}$. Direct fusion is inappropriate because the former is indexed by historical time and the latter by forecast lead. A learned horizon projection Q maps the observation history to lead-conditioned station states, and $P_{K}$ denotes a learnable aggregation along the *K*-neighbor axis. The aligned features are then fused as

(6) $$Z^{t}={\mathrm{MLP}}_{t}\left(Q\left(H^{\mathrm{obs}}\right)∥P_{K}\left(H^{\mathrm{nwp}}\right)\right)\in R^{T_{\mathrm{out}}\times N\times D}.$$

Thus, $Z^{t}$ represents how the future NWP trajectory should be corrected at each lead in light of the station’s recent evolution. Observations condition the physically guided future trajectory rather than being used to extrapolate it independently.

#### 2.3.2. Coordinate-Conditioned Adaptive Station Graph

The aligned temporal states are initially station-specific, although meteorological disturbances extend across multiple sites. TF-STNet therefore propagates them over an adaptive station graph. Coordinates S provide a stable geographic prior, while separate source and target embeddings allow learned dependence to depart from a symmetric distance kernel. This flexibility accommodates directional relationships induced by terrain, coastlines, and prevailing flow. The embeddings are

(7) $$U_{\mathrm{src}}={\mathrm{MLP}}_{\mathrm{src}}\left(S\right),\;U_{\mathrm{tgt}}={\mathrm{MLP}}_{\mathrm{tgt}}\left(S\right),\;U_{\mathrm{src}},U_{\mathrm{tgt}}\in R^{N\times D_{g}},$$

where $D_{g}$ is the graph-embedding dimension. Their compatibility scores define a directed, row-normalized adaptive adjacency matrix,

(8) $$A={\mathrm{Softmax}}_{\mathrm{row}}\left(\mathrm{ReLU}(U_{\mathrm{src}}U_{\mathrm{tgt}}^{⊤})\right)\in R^{N\times N}.$$

Separate source and target embeddings permit $A_{ij}\neq A_{ji}$ for station indices *i* and *j*. At each forecast lead τ, the graph layer combines messages from related stations with a station-wise residual transformation:

(9) $$H_{\tau }^{s}=\phi \left(AZ_{\tau }^{t}W_{g}+Z_{\tau }^{t}W_{\mathrm{res}}\right),$$

where $H^{s}\in R^{T_{\mathrm{out}}\times N\times D}$ denotes the spatially propagated hidden states; $W_{g},W_{\mathrm{res}}\in R^{D\times D}$ are learnable graph-message and residual projections, respectively; and ϕ is a nonlinear activation. The residual term limits excessive graph smoothing and retains station-specific behavior. A temporal prediction head then produces the time-domain candidate

(10) $${\hat{Y}}^{t}={\mathrm{Head}}_{\mathrm{time}}\left(H^{s}\right)\in R^{T_{\mathrm{out}}\times N\times C}.$$

### 2.4. Frequency-Domain Pathway

#### 2.4.1. Spectral Encoding

The time-domain pathway emphasizes temporal order and lead-specific transitions but does not explicitly isolate oscillatory structure across the complete horizon. The frequency-domain pathway provides this complementary view using the same local NWP tensor $E^{K}$, thereby remaining anchored to the physically generated future guidance. By reindexing the forecast leads as $\tau \in \{0,\ldots ,T_{\mathrm{out}}-1\}$ for the transform, the discrete Fourier coefficient for each station–neighbor–variable tuple (i,k,c) is

(11) $$F_{\omega ,i,k,c}=\sum_{\tau =0}^{T_{\mathrm{out}}-1}E_{\tau ,i,k,c}^{K}\exp\left(-j\frac{2\pi \omega \tau }{T_{\mathrm{out}}}\right),$$

where ω is the frequency-bin index, $\Omega =\lfloor T_{\mathrm{out}}/2\rfloor +1$ is the number of retained bins, and $j^{2}=-1$. Thus, ω∈{0,…,Ω−1}. Only the nonredundant coefficients of the real-valued trajectory are retained. A bounded spectral gate $w_{f}\in R^{\Omega }$ learns the relative relevance of these frequency bins:

(12) $${\tilde{F}}_{\omega ,i,k,c}=F_{\omega ,i,k,c}\;\sigma \left(w_{f,\omega }\right).$$

The gate is shared across stations and local neighbors within each regional model, encouraging region-level spectral selection rather than sample-limited station-specific filtering. The sigmoid bounds each multiplier, preventing unstable amplification while attenuating weakly informative frequency bins.

Collecting the coefficients into $\tilde{F}\in C^{\Omega \times N\times K\times C}$, the gated spectrum is decomposed into amplitude and phase:

(13) M=|F˜|,Φ=atan2ImF˜,ReF˜,

where $M,\Phi \in R^{\Omega \times N\times K\times C}$. Amplitude describes the strength of an oscillatory component, whereas phase locates it relative to the forecast origin. Treating them separately prevents a neighboring cell with realistic variability but incorrect timing from being considered equivalent to one with accurate timing but distorted variability. This distinction is important for fronts, sea-breeze cycles, and terrain-modulated responses whose timing can vary across nearby grid cells.

#### 2.4.2. Amplitude–Phase Attention and Frequency-Domain Prediction

Amplitude and phase are projected independently into a common hidden dimension, allowing each component to learn a distinct nonlinear representation before neighborhood aggregation:

(14) $$Z^{M}={\mathrm{MLP}}_{M}\left(M\right),\;Z^{\Phi }={\mathrm{MLP}}_{\Phi }\left(\Phi \right),\;Z^{M},Z^{\Phi }\in R^{\Omega \times N\times K\times D}.$$

For q∈{M,Φ} and fixed (ω,i), let $Z_{\omega ,i}^{q}\in R^{K\times D}$ denote the hidden vectors of the *K* neighboring NWP cells. Scaled dot-product attention is then applied only over this local-neighbor axis:

$$\begin{array}{cc} Q_{\omega ,i}^{q} & =Z_{\omega ,i}^{q}W_{Q}^{q},\;K_{\omega ,i}^{q}=Z_{\omega ,i}^{q}W_{K}^{q},\;V_{\omega ,i}^{q}=Z_{\omega ,i}^{q}W_{V}^{q}, \end{array}$$

(15) $$\begin{array}{cc} O_{\omega ,i}^{q} & ={\mathrm{Attn}}_{K}\left(Z_{\omega ,i}^{q}\right)\;\;\;\;\;\;\;\;\;\;\;\;\; \end{array}$$

(16) $$\begin{array}{cc} \; & ={\mathrm{Softmax}}_{K}\left(\frac{Q_{\omega ,i}^{q}{\left(K_{\omega ,i}^{q}\right)}^{⊤}}{\sqrt{D}}\right)V_{\omega ,i}^{q},\;\;\;\;\; \end{array}$$

where $W_{Q}^{q},W_{K}^{q},W_{V}^{q}\in R^{D\times D}$ are component-specific query, key, and value projections, respectively. For fixed (ω,i,q), the resulting $Q_{\omega ,i}^{q}$, $K_{\omega ,i}^{q}$, $V_{\omega ,i}^{q}$, and $O_{\omega ,i}^{q}$ all belong to $R^{K\times D}$, and ${\mathrm{Softmax}}_{K}$ normalizes each query over the *K* neighboring cells. Attention is therefore restricted to the local-neighbor axis and conditioned on frequency and station, so the preferred NWP cell may vary across locations and oscillatory scales. Consequently, $O^{M}$ can favor cells that better represent variation strength, while $O^{\Phi }$ can favor different cells with more consistent timing. Component-specific parameters preserve this distinction until the two forms of evidence are combined.

The two attention outputs are concatenated and mapped to a joint spectral representation:

(17) $$H^{f}=P_{K}\left[{\mathrm{MLP}}_{f}\left(O^{M}∥O^{\Phi }\right)\right].$$

Here, the frequency-pathway instance of $P_{K}$ aggregates the attended neighbor axis, yielding $H^{f}\in R^{\Omega \times N\times D}$. A frequency prediction head then performs learned spectral-to-horizon reconstruction and variable projection:

(18) $${\hat{Y}}^{f}={\mathrm{Head}}_{\mathrm{freq}}\left(H^{f}\right)\in R^{T_{\mathrm{out}}\times N\times C}.$$

Because separate attention and nonlinear fusion mix evidence from multiple neighboring trajectories, $H^{f}$ is not the spectrum of a single physical signal and cannot be reconstructed by a direct inverse Fourier transform. The learned head instead maps the selected spectral evidence to the lead-resolved frequency-domain candidate.

### 2.5. Prediction-Level Time–Frequency Fusion and Training Objective

The two pathway outputs are correction candidates with the same $T_{\mathrm{out}}\times N\times C$ station-by-variable forecast structure. They are fused as

(19) $$\hat{Y}={\mathrm{MLP}}_{o}\left({\hat{Y}}^{t}∥{\hat{Y}}^{f}\right).$$

Fusion at the prediction level combines tensors with the same physical interpretation and avoids imposing arbitrary alignment between lead-indexed temporal states and frequency-indexed features. It also preserves pathway specialization while ${\mathrm{MLP}}_{o}$ learns a nonlinear combination that can vary across variables, stations, and forecast leads. All modules, from local extraction to the final output head, are trained end to end using mean absolute error (MAE):

(20) $$L_{\mathrm{MAE}}=\frac{1}{T_{\mathrm{out}}NC}\sum_{\tau =1}^{T_{\mathrm{out}}}\sum_{i=1}^{N}\sum_{c=1}^{C}\left|{\hat{Y}}_{\tau ,i,c}-Y_{\tau ,i,c}\right|.$$

MAE matches the primary evaluation metric and is less sensitive than squared loss to a small number of large residuals.

## 3. Experimental Design

### 3.1. Study Regions and Data

**Figure 3:**
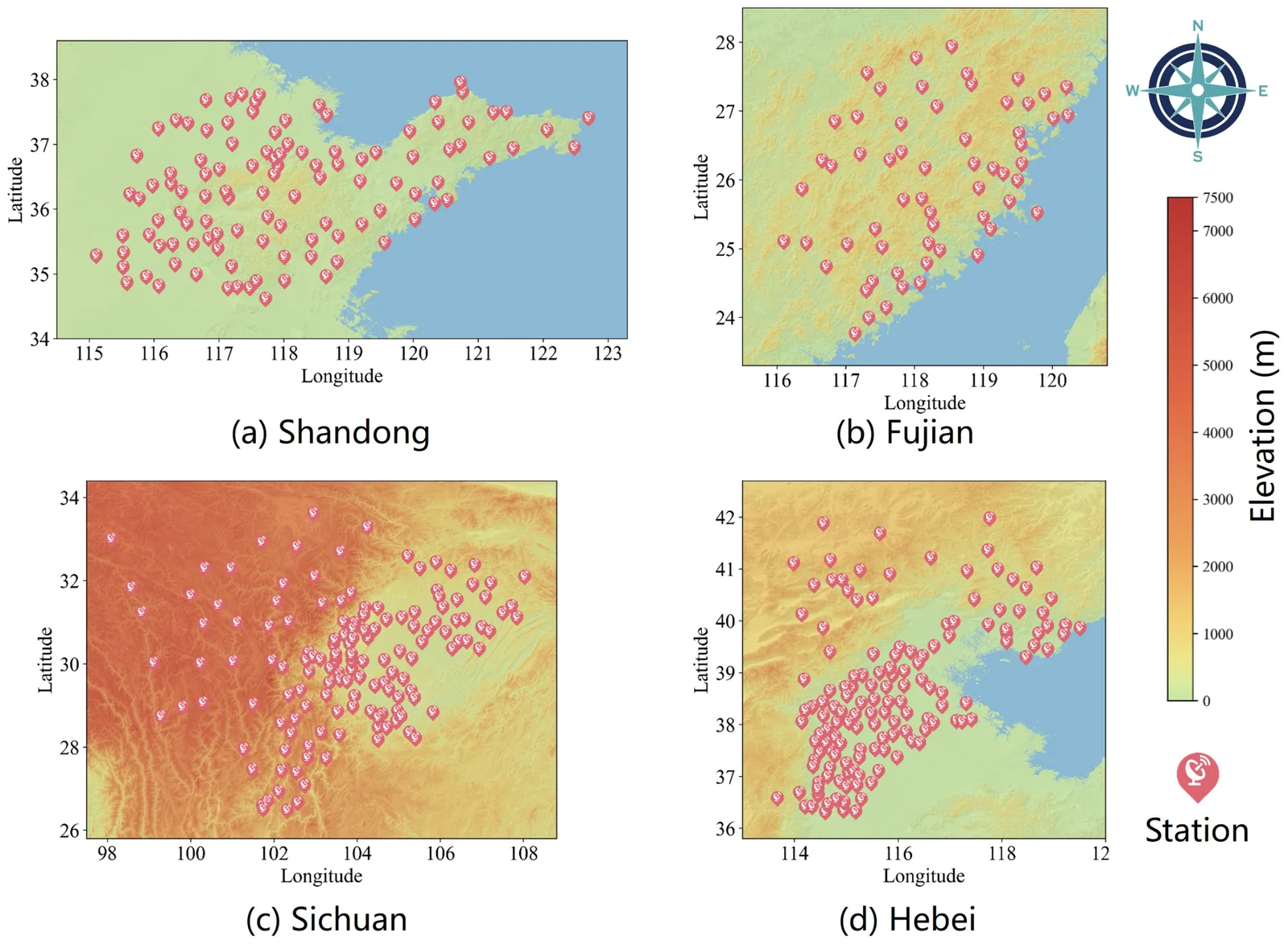
Terrain and station distributions in the four study regions: (**a**) Shandong, (**b**) Fujian, (**c**) Sichuan, and (**d**) Hebei. Background color denotes elevation, and markers indicate meteorological stations.

The evaluation uses hourly station observations and corresponding NWP forecasts from 1 May 2024 to 30 November 2025. The dataset covers 455 national meteorological stations in four geographically distinct regions of China: Shandong (109 stations; 34–38° N, 115–123° E), Fujian (60 stations; 23–28° N, 116–121° E), Sichuan (145 stations; 26–34° N, 97–109° E), and Hebei (141 stations; 36–42° N, 113–120° E). The NWP fields have a horizontal resolution of 0.1° and are stored as regional tensors containing 65×68, 85×42, 50×50, and 113×85 grid cells, respectively. As shown in Figure 3, these regions differ substantially in terrain and station density, providing diverse conditions for evaluating grid-to-station correction.

Table 1 compares the observed and raw NWP distributions. The discrepancies vary substantially across both variables and regions. Mean NWP wind speed exceeds the observed mean by 1.47, 1.21, and 1.16 m s^−1^ in Shandong, Fujian, and Hebei, respectively, but is nearly unbiased in Sichuan. Pressure exhibits a different pattern: the Sichuan observation and NWP means are 888 and 769 hPa, respectively, a 119 hPa discrepancy. This unusually large offset is consistent with the sensitivity of pressure to differences between grid-cell terrain height and station elevation; it helps explain why pressure shows the largest correction gains in this study and why raw pressure should not be compared across regions without considering elevation. In Hebei, raw NWP pressure is lower on average and substantially more variable than the observations. These additive, dispersion, and region-dependent errors motivate a conditional station-correction model rather than a uniform adjustment.

### 3.2. Quality Control and Sample Construction

Observation and NWP timestamps, time zones, and physical units were first harmonized. Missing observation intervals of at most 3 h were filled by temporal interpolation, whereas windows containing longer gaps were excluded. Potentially implausible values were identified using variable-specific physical ranges and a *z*-score rule and then checked against adjacent times and neighboring stations. NWP forecasts were aligned with station observations by initialization time, lead time, and valid time. The raw element-wise missing rates recorded before this quality-control step were 3.0–3.2% in training, approximately 1.0% in validation, and 2.1–2.4% in testing (Table 2). Because the retained quality-control records provide element-wise rather than window-level counts, the exact fraction of windows removed by the long-gap rule cannot be reconstructed. The records also do not indicate whether an interpolated target within a retained window was used as a scored label. We therefore report this detail as unresolved rather than inferring an undocumented policy. At evaluation, any target position that remains unavailable is excluded from both the absolute-error sum and the denominator. With $v_{m}\in \left\{0,1\right\}$ indicating whether target position *m* is available, the masked metric is ${\mathrm{MAE}}_{\mathrm{mask}}=\sum_{m}v_{m}\left|{\hat{y}}_{m}-y_{m}\right|/\sum_{m}v_{m}$.

Samples were partitioned chronologically into 9752 training, 2098 validation, and 2098 test windows, corresponding to approximately 70%, 15%, and 15% of the data. Preserving temporal order prevents information from later periods from entering model training. This split tests temporal generalization within the four regional station networks; it does not test transfer to an unseen station, region, or climate regime. Consistent with the problem formulation, each sample contains 24 h of historical station observations, 24 h of future NWP guidance, and 24 h of future station targets. Normalization parameters were estimated from the training partition and applied unchanged to the validation and test partitions.

### 3.3. Baselines, Model Selection, and Evaluation Metrics

The evaluation compares TF-STNet with raw NWP and five learned baselines: Transformer [21], PatchTST [23], DLinear [24], Graph WaveNet (GWNet) [17], and MegaCRN [18]. Transformer, PatchTST, and DLinear are representative sequence-modeling baselines, whereas GWNet and MegaCRN are graph-based spatiotemporal forecasters. For a forecast issued at time *t*, all learned methods use station observations from t−23 to *t*, future NWP guidance from t+1 to t+24, and station targets over the same t+1 to t+24 period. Every learned baseline is evaluated on the same chronological partitions and forecast cases. The sequence baselines do not use explicit coordinate information, whereas the graph-based baselines and TF-STNet incorporate geographic relationships through their respective graph representations. In TF-STNet, coordinate embeddings are used to construct the adaptive graph. The comparison therefore equalizes the principal data sources and temporal support while retaining architecture-specific spatial representations. Raw NWP is evaluated without any learned correction. GraphCast and related global foundation models are not treated as drop-in baselines because they require global, multilevel initial states and produce gridded atmospheric forecasts; replacing the supplied regional NWP would change the upstream forecast system rather than test NWP-to-station post-processing. Their omission is therefore a scope limitation, not a claim that they are inferior.

Each method was trained and evaluated with random seeds 2026, 2025, 2024, 2023, and 2022 using the same split and evaluation procedure; we report the arithmetic mean and standard deviation across runs. For TF-STNet, *K* and *D* were selected independently by one-factor-at-a-time validation sweeps: K∈{1,…,10} and D∈{16,32,64,128,256}. These ranges span a single nearest cell through a broad local neighborhood and cover small to moderately overparameterized hidden representations. The task-specific selections are reported in Section 4.7.

Table 3 summarizes the fixed optimization, checkpoint, initialization, and seed settings. The public repository at https://github.com/gyla1993/TS-STNet_source_code (accessed on 3 September 2026) provides the model implementation, configuration files, and a wind-speed training and inference example.

Table 4 summarizes the input availability and temporal alignment for the compared methods.

The primary evaluation metric is MAE:

(21) $$\mathrm{MAE}=\frac{1}{M}\sum_{m=1}^{M}\left|{\hat{y}}_{m}-y_{m}\right|,$$

where *M* is the total number of scalar predictions across all test windows, forecast leads, and stations within one region–variable task; *m* indexes those predictions, and ${\hat{y}}_{m}$ and $y_{m}$ are the corresponding prediction and observation. For the multi-seed comparison, MAE is complemented by the Pearson correlation coefficient (PCC), which measures the linear association between predictions and observations:

(22) $$\mathrm{PCC}=\frac{\sum_{m}\left({\hat{y}}_{m}-\bar{\hat{y}}\right)\left(y_{m}-\bar{y}\right)}{\sqrt{\sum_{m}{\left({\hat{y}}_{m}-\bar{\hat{y}}\right)}^{2}}\sqrt{\sum_{m}{\left(y_{m}-\bar{y}\right)}^{2}}},$$

where $\bar{\hat{y}}$ and $\bar{y}$ are the means of predictions and observations over the same valid positions. Because MAE has different units and scales across variables, aggregate performance for method *q* in region *r* is summarized by normalized MAE (NMAE):

(23) $${\mathrm{NMAE}}_{q,r}=\frac{1}{C}\sum_{c=1}^{C}\frac{{\mathrm{MAE}}_{q,r,c}}{{\mathrm{MAE}}_{\mathrm{NWP},r,c}}.$$

Here, *q* indexes the forecasting method, *r* the region, and *c* the meteorological variable; ${\mathrm{MAE}}_{\mathrm{NWP},r,c}$ is the raw NWP MAE for region *r* and variable *c*. Raw NWP therefore has an NMAE of 1 by definition, while lower values indicate larger aggregate correction gains. The four-region mean NMAE reported in the Results averages Equation (23) across the four study regions.

## 4. Results and Discussion

### 4.1. Improvement over Raw NWP

This analysis assesses whether TF-STNet consistently corrects raw NWP across meteorological variables and geographic regions, rather than deriving its overall gain from a small number of favorable tasks. We report the five-run mean and standard deviation for each method and examine the percentage reduction in MAE for each of the 16 region–variable combinations.

Table 5 provides the updated five-run results. TF-STNet has the lowest mean MAE in 15 of the 16 tasks and the highest mean PCC in 15 tasks. The only MAE exception is Shandong–temperature (0.485 ± 0.017), for which MegaCRN (0.430 ± 0.082) has lower errors. The pressure MAE reductions relative to the best learned comparator are 16.2% in Shandong, 26.6% in Fujian, 17.0% in Sichuan, and 57.4% in Hebei. Standard deviations are small for TF-STNet in most tasks, whereas several baselines show larger variation in pressure and temperature.

In summary, the multi-seed analysis supports broad but not universal superiority over the learned baselines. The raw NWP reduction remains strongly variable-dependent, with pressure showing the largest gains because its grid-to-station offset is especially systematic; temperature, particularly in Shandong, is less uniformly correctable.

**Figure 4:**
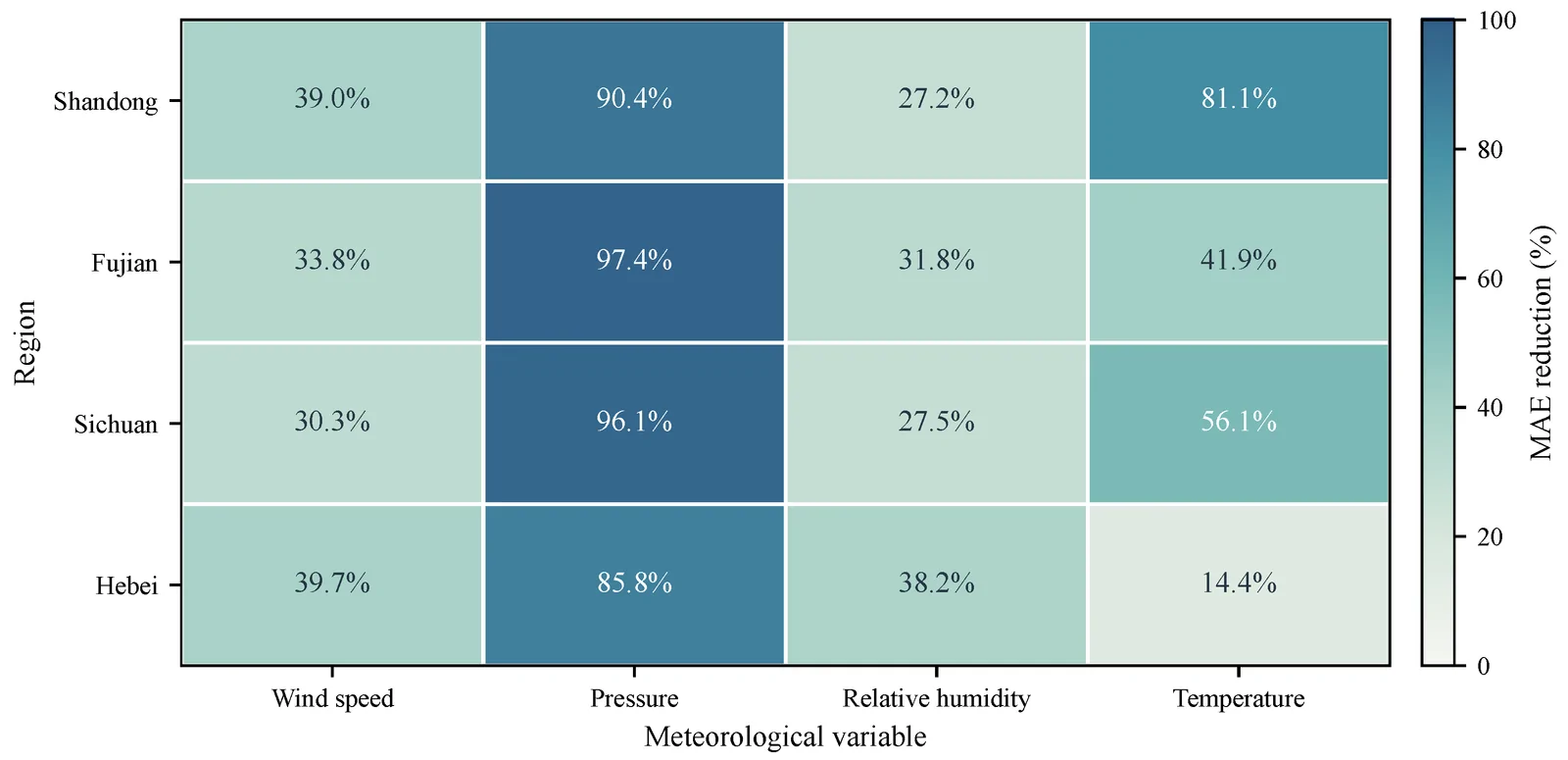
Percentage reduction in five-seed mean MAE achieved by TF-STNet relative to raw NWP for each region–variable task. Higher values indicate greater improvement; negative values would indicate degradation.

Figure 4 visualizes the corresponding task-wise MAE reductions relative to raw NWP, making the strong pressure improvements and the greater regional variation in temperature readily apparent.

### 4.2. Comparison with Forecasting Baselines

Table 6 summarizes NMAE computed from the five-run mean MAE values. TF-STNet achieves the lowest NMAE in every region and a four-region mean of 0.481. After correction of the Shandong–temperature entry, GWNet is the strongest aggregate comparator, with a mean of 0.531, so the relative reduction is 9.4%; MegaCRN has a corrected four-region mean of 0.558. The regional NMAE reductions relative to MegaCRN are 10.7% in Shandong, 9.1% in Fujian, 5.4% in Sichuan, and 24.2% in Hebei. These aggregate values should be read together with the task-wise standard deviations in Table 5; NMAE is a descriptive scale-normalized summary, not an additional independent test.

Although the NMAE of TF-STNet ranges from 0.406 in Shandong to 0.555 in Hebei, it ranks first in each region. Thus, the aggregate improvement is observed across all four study areas rather than being driven by a single region.

The four-region mean NMAE table provides an aggregate comparison of the five-run means. TF-STNet has the lowest four-region mean MAE for all four variables, although Table 5 shows that this aggregate result does not imply the lowest MAE in every individual task. In particular, Shandong–temperature remains better served by several baselines. Thus, the aggregate advantage is not driven solely by the highly correctable pressure field, but the temperature result also cautions against claiming universal task-wise dominance.

**Figure 5:**
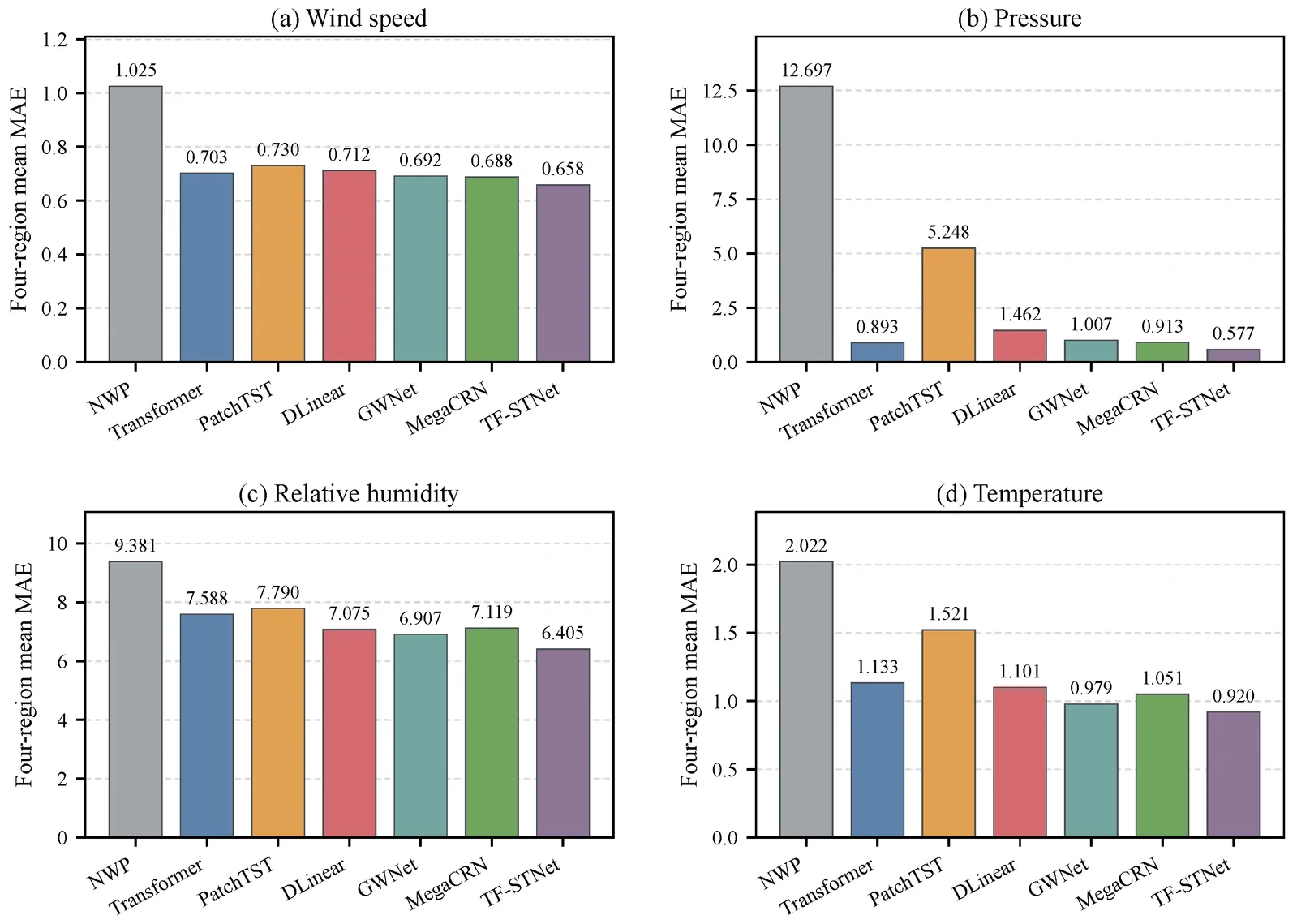
Four-region mean MAE of raw NWP and the learned forecasting methods, computed from the five-seed means, for (**a**) wind speed, (**b**) pressure, (**c**) relative humidity, and (**d**) temperature. Lower values indicate better performance.

GWNet and MegaCRN generally outperform the sequence-only baselines, a pattern consistent with the value of explicit spatial modeling. PatchTST is less stable in pressure and temperature, as indicated by its larger five-run deviations. The further improvement achieved by TF-STNet, together with the frequency-pathway ablation below, suggests that adaptive station graphs do not capture all useful structure in local NWP trajectories and that complementary spectral modeling provides additional information. Figure 5 complements the regional NMAE summary by comparing the four-region mean MAE of all methods separately for each meteorological variable.

### 4.3. Simple Pressure MOS Baselines

The large pressure correction gains could otherwise be interpreted as removal of an almost constant offset. We therefore compare TF-STNet with two deliberately simple model-output-statistics (MOS) baselines: mean-bias correction, $\hat{y}=x+b$ with $b=\mathrm{mean}(y_{\mathrm{train}}-x_{\mathrm{train}})$, and univariate linear MOS, $\hat{y}=ax+b$ fitted on the training set. Both methods use only the local NWP pressure value and are evaluated on the same test cases as TF-STNet. This auxiliary analysis uses the reference seed 2026. Table 7 shows that TF-STNet has lower MAE in all four regions. The reduction relative to linear MOS is 27.3–84.3%, and the reduction relative to mean-bias correction is 44.3–86.5%. Thus, subtracting a single climatological bias does not explain the full pressure improvement; the result is consistent with additional contributions from station history, local spatial context, and the nonlinear lead-dependent mapping.

### 4.4. Forecast-Lead and Extreme-Event Evaluation

**Figure 6:**
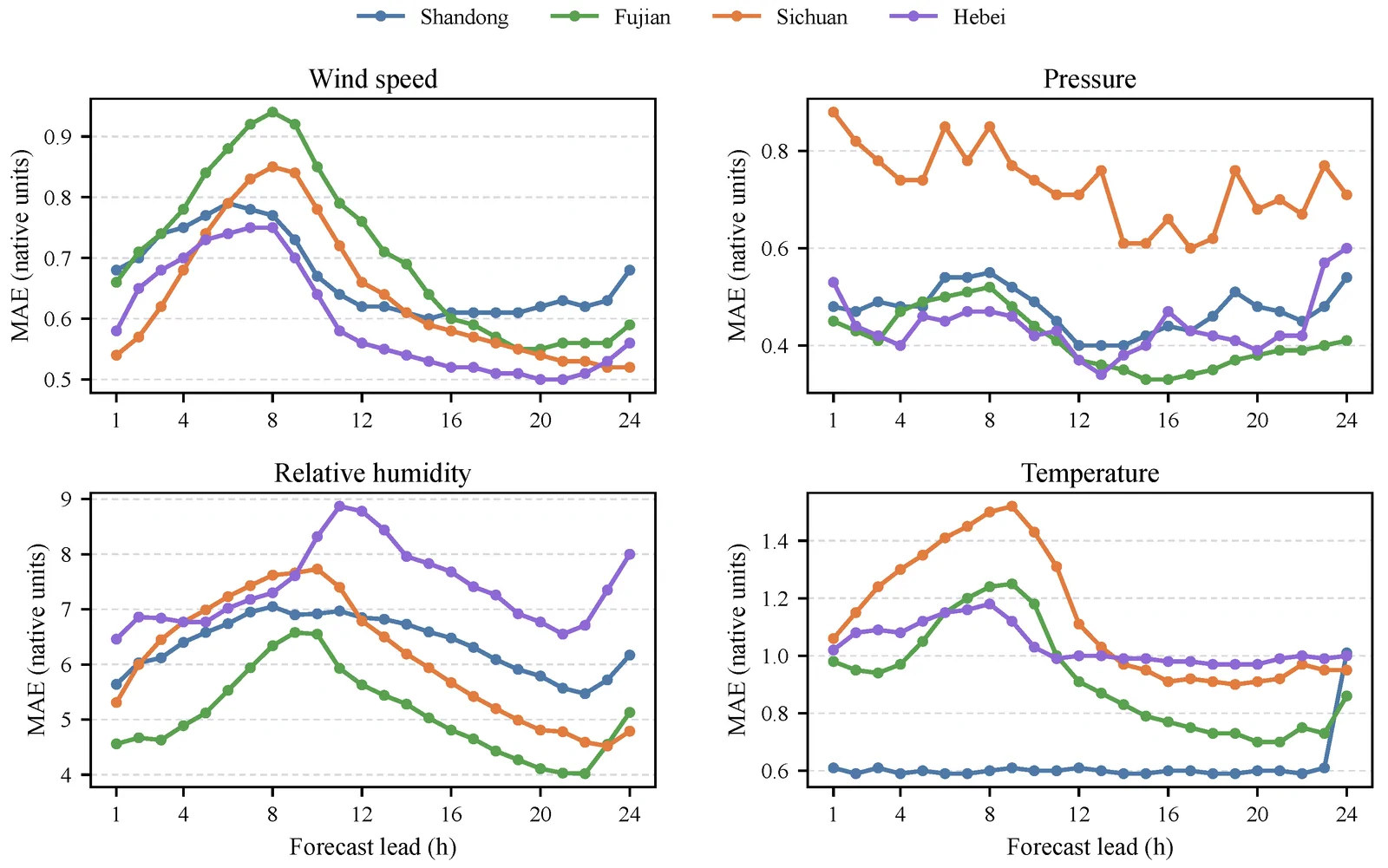
TF-STNet test-set MAE at each forecast lead from 1 to 24 h. Each panel is a meteorological variable, and each curve is one region; the independent y-scales preserve visibility across variables with different units and error magnitudes.

Aggregate MAE can conceal difficult leads or tail events. We therefore grouped the reference-seed test predictions by lead from 1 to 24 h and computed MAE separately at each lead. Figure 6 visualizes all 24 lead-specific errors, while Table 8 reports their mean and the leads attaining the minimum and maximum. The errors are non-monotonic: peaks occur around 6–11 h for several wind-speed, relative-humidity, and temperature series, while Shandong–temperature and Hebei–pressure increase near the end of the horizon. The model produces predictions throughout the 24-h horizon, but the lead-specific peaks identify the forecast periods with the largest errors. Because comparator outputs were not retained by lead, this analysis characterizes TF-STNet but does not establish a lead-wise advantage over the baselines.

For tail verification, a high-wind event is a test position at which wind speed exceeds the training-set 90th percentile; a rapid-change event is a position at which the absolute change from the adjacent time exceeds the corresponding training-set 90th percentile. The thresholds are determined from training data only, and raw NWP, MegaCRN, and TF-STNet are evaluated on identical event sets using the reference seed 2026 for the learned models. Table 9 shows that TF-STNet has lower MAE than MegaCRN in all eight cases and than raw NWP in seven. The sole exception is the Hebei–high-wind event, for which the raw NWP and TF-STNet MAE are 1.427 and 1.523, respectively, although TF-STNet remains more accurate than MegaCRN (1.687). Across the eight cases, the average improvements are 17.5% relative to raw NWP and 6.2% relative to MegaCRN. These results indicate improved performance for the tested wind events without implying universal superiority under rare conditions.

### 4.5. Computational Efficiency and Reproducibility

Efficiency was measured on the same NVIDIA GeForce RTX 3080 Ti GPU with batch size 32. Table 10 reports wall-clock time for one training batch, one inference batch, and the number of trainable parameters. TF-STNet is consistently faster than MegaCRN (29.4–54.4% lower training time and 25.0–65.8% lower inference time) and has a similar though not identical cost to GWNet. It uses more parameters (0.837–0.941 million) than the two graph baselines, so the timing advantage is due to the measured implementation rather than a smaller model. The complete optimization, initialization, checkpoint, and seed settings are reported in Section 3.3. The public repository provides the model implementation, configuration files, and a wind-speed training and inference example.

### 4.6. Ablation Study

Four ablations separate the effects of input sources from those of the two representation pathways. The w/o NWP and w/o Obs. variants remove future NWP and historical observations, respectively; w/o Time and w/o Freq. remove the corresponding pathway while retaining the remaining inputs where applicable. Table 11 reports relative MAE, computed for each region by averaging the four ratios between an ablation’s variable-wise MAE and that of the complete model. TF-STNet is normalized to 1.0, so values above 1 indicate degradation. The ablation analysis uses the reference seed 2026, whereas the main comparison in Table 5 reports the five-seed robustness analysis.

Removing the time-domain pathway causes the largest mean degradation, increasing relative MAE to 1.885, followed by removal of NWP at 1.819. The latter nearly doubles the error because it removes guidance over the future horizon, consistent with the intended role of TF-STNet as an NWP post-processor rather than an autoregressive station forecaster. The Sichuan w/o-Time value (2.899) is neither a percentage nor an absolute MAE; it is the mean of four variable-wise ratios. It is amplified by the pressure component, for which losing lead-resolved temporal states leaves a large terrain-related level error relative to the small complete-model MAE. Removing the frequency-domain pathway raises the mean relative MAE to 1.396, indicating that spectral amplitude and phase provide information not recovered by the time-domain pathway. Removing observations produces the smallest but still consistent degradation, to 1.154, quantifying the value of recent measurements for local state calibration. Together, these results indicate that future NWP and the time-domain pathway provide the largest aggregate contributions, while observations and frequency-domain learning provide complementary gains.

**Figure 7:**
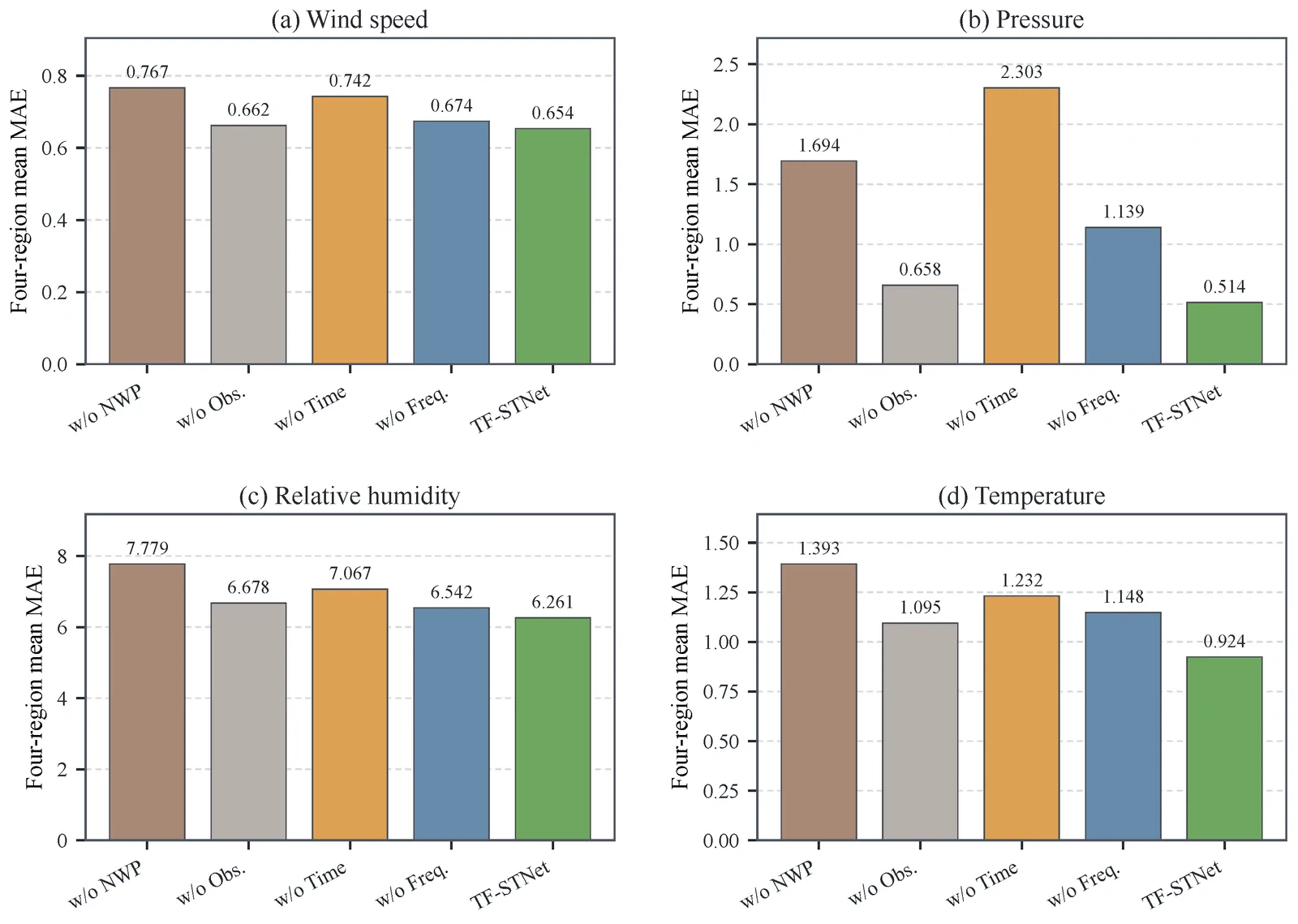
Four-region mean MAE of the ablation variants for (**a**) wind speed, (**b**) pressure, (**c**) relative humidity, and (**d**) temperature. Lower values indicate better performance.

Figure 7 presents the corresponding four-region mean MAE by variable. Complete TF-STNet achieves the lowest value in every panel. Without NWP, mean MAE increases to 0.767, 1.694, 7.779, and 1.393 for wind speed, pressure, relative humidity, and temperature, respectively. Removing the time-domain pathway raises pressure MAE to 2.303, 4.48 times that of the complete model, while removing the frequency-domain pathway raises pressure and temperature MAE to 1.139 and 1.148. Because the figure reports cross-region means, it characterizes average pathway complementarity; Table 11 provides the corresponding region-specific behavior.

Pressure provides the clearest evidence of pathway complementarity: removing either pathway markedly increases its mean MAE, so the improvement cannot be attributed to adaptive graph propagation or spectral processing alone. The frequency-domain pathway also contributes substantially to temperature, for which its removal increases the mean MAE by 24.2%. Its smaller effect on wind speed and relative humidity may reflect their more intermittent, less strongly periodic behavior over a 24-h horizon. In contrast, pressure and temperature contain stronger low-frequency or diurnal structure, giving amplitude–phase separation more opportunity to distinguish systematic distortions. This explanation is an interpretation of the observed ablation pattern, not a causal claim about atmospheric dynamics. Although the regional penalties vary in magnitude, every ablation performs worse than the complete TF-STNet in every region, indicating that the pathway benefits are not artifacts of cross-region averaging.

### 4.7. Hyperparameter Sensitivity

**Figure 8:**
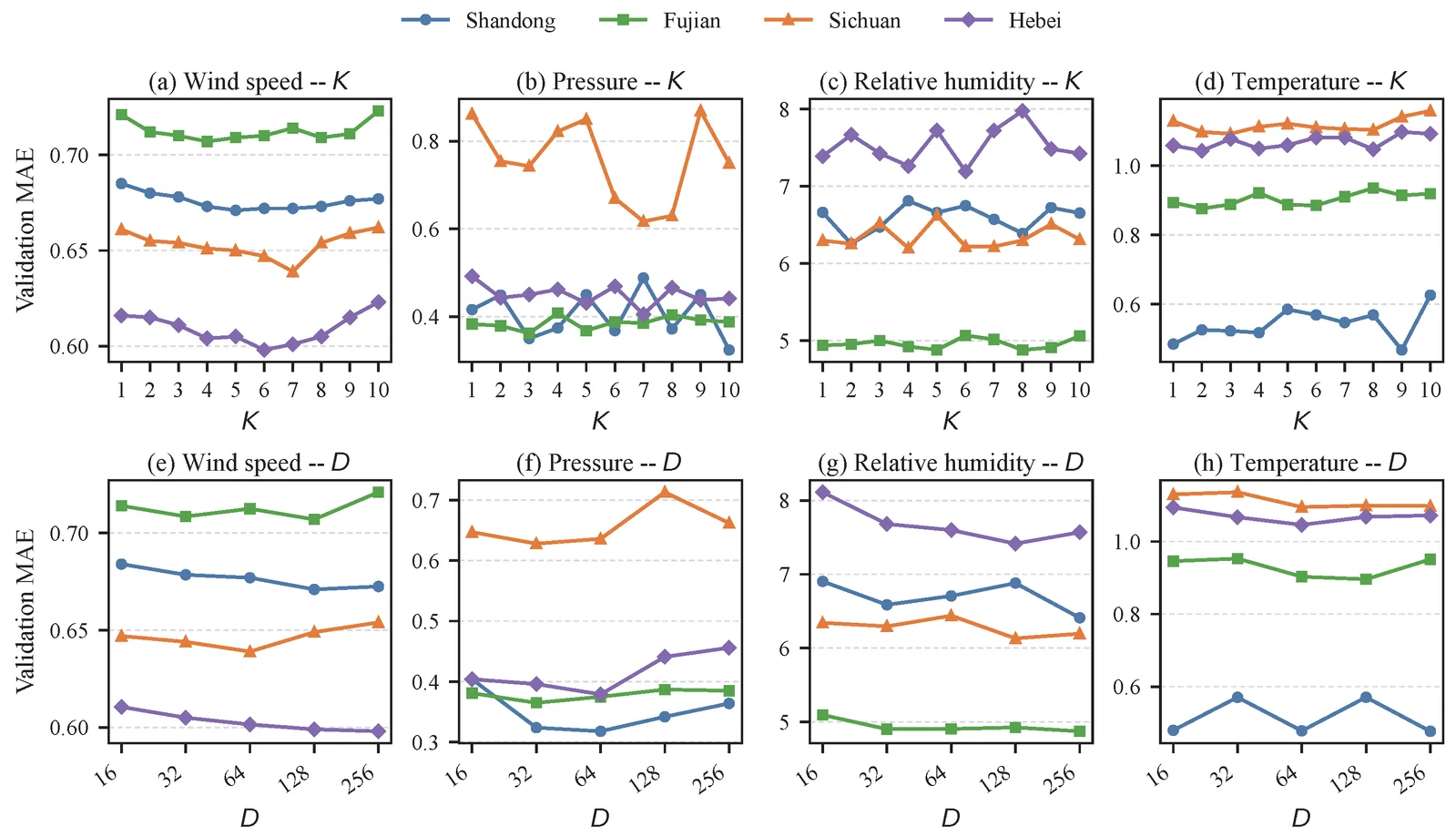
Unified validation-MAE sensitivity curves for all four meteorological variables. The top row varies the number of local NWP neighbors *K* and the bottom row varies the hidden dimension *D*; each curve corresponds to one region. Lower values indicate better performance within a panel.

Figure 8 consolidates the one-factor-at-a-time validation sweeps for all four variables. The top row varies the number of retained NWP neighbors *K* from 1 to 10, and the bottom row varies the hidden dimension *D* over 16, 32, 64, 128, and 256. Curves are plotted separately for each region because the error scales differ across variables; comparisons should therefore be made within a panel rather than between panels.

The response to *K* is non-monotonic and variable-dependent. For wind speed, the minima occur at K=5, 4, 7, and 6 for Shandong, Fujian, Sichuan, and Hebei, respectively. The pressure optima shift to K=10, 3, 7, and 7; temperature favors K=9, 2, 3, and 2; and relative humidity favors K=2, 8, 4, and 6. This pattern indicates that a larger neighborhood is not uniformly beneficial: too few cells omit local context, whereas too many can introduce weakly related trajectories. The preferred *D* values are similarly heterogeneous. Wind speed selects D=128, 128, 64, and 256 across the four regions, while pressure selects 64, 32, 32, and 64; temperature selects 256, 128, 64, and 64; and relative humidity selects 256, 256, 128, and 128. The D=16 candidate is never optimal, but increasing capacity beyond the selected value does not yield monotonic gains. Table 12 lists every selected configuration and validation MAE. Together, Figure 8 and Table 12 show why *K* and *D* must be selected per region and variable rather than fixed globally.

## 5. Conclusions

This study presents TF-STNet for translating gridded NWP forecasts into station-level meteorological trajectories. Its source-aware time-domain pathway aligns recent observations with future NWP and propagates lead-resolved states over an adaptive station graph. Its frequency-domain pathway evaluates local NWP neighborhoods through spectral weighting and separate amplitude and phase attention. Prediction-level fusion combines the two candidate forecasts while preserving the distinct role of each representation.

Across five independent runs and 16 region–variable tasks spanning 455 stations in Hebei, Shandong, Fujian, and Sichuan, TF-STNet has the lowest mean MAE in 15 tasks and the highest mean PCC in 15 tasks. It achieves a four-region mean NMAE of 0.481, has lower pressure MAE than the simple MOS corrections in all four regions, and has lower wind-event MAE than MegaCRN in all eight tested cases. The ablation results indicate that future NWP, recent observations, and both pathways provide complementary information, with future NWP and the time-domain pathway making the largest aggregate contributions.

The evidence also defines the boundaries of these conclusions. The chronological split assesses temporal generalization within the four existing station networks, not transfer to unseen stations or regions. The evaluation is deterministic, so probabilistic calibration is not established. Event tests cover only high wind and rapid wind changes; moreover, the Hebei–high-wind case and the Shandong–temperature task show that the method is not uniformly the best. The available quality-control summary does not provide the exact fraction of windows excluded by the long-gap rule or establish whether interpolated targets were retained for scoring. Future work should consider probabilistic outputs, transfer to unseen stations and regions, broader event-stratified verification, and comparisons across alternative upstream forecast systems.

## Figures and Tables

**Table 1:** Observed and raw NWP statistics in the four study regions (mean ± standard deviation).

Region	Source	Wind Speed (m s^−1^)	Pressure (hPa)	Relative Humidity (%)	Temperature (°C)
Shandong	Observation	2.36±1.72	1004.00±19.49	63.99±24.00	17.70±10.58
	NWP	3.83±2.44	1008.00±13.34	62.20±22.78	17.70±10.00
Fujian	Observation	2.14±1.76	986.00±29.60	75.90±17.66	22.00±7.57
	NWP	3.35±3.54	976.00±32.09	76.90±17.35	21.50±7.26
Sichuan	Observation	1.69±1.30	888.00±32.42	72.40±21.24	17.80±9.21
	NWP	1.68±1.30	769.00±43.19	70.75±22.25	11.06±10.44
Hebei	Observation	1.93±1.57	993.00±37.78	60.70±25.46	16.00±11.53
	NWP	3.09±2.11	953.00±63.65	56.70±24.04	13.70±11.87

**Table 2:** Raw observation missing rates before interpolation or long-gap exclusion. Rates are element-wise percentages pooled over the four variables; the within-region rates were reported as consistent across variables.

Region	Training	Validation	Test
Fujian	3.0%	1.0%	2.1%
Shandong	3.1%	1.0%	2.2%
Hebei	3.2%	1.0%	2.3–2.4%
Sichuan	3.1%	1.0%	2.4%

**Table 3:** Fixed training configuration. Settings were held constant across regions, variables, and experimental analyses unless otherwise noted.

Item	Setting
Optimizer	Adam
Batch size	32
Training duration	300 epochs
Early stopping	Not used
Checkpoint rule	Retain the checkpoint with the lowest validation MAE after the completed 300-epoch run
Initial learning rate	0.001
Learning-rate schedule	None
Weight decay	0.0001
Dropout	0.1
Frequency-module initialization	Xavier uniform for linear weights; zero for linear biases; one for learnable frequency weights
Other-layer initialization	PyTorch 2.3.1 defaults
Reference/auxiliary seed	2026
Robustness seeds	2026, 2025, 2024, 2023, and 2022
Variable task selection	*K* and *D* selected independently by the lowest validation MAE; all other settings fixed

**Table 4:** Information available to each method in the reported comparison. A checkmark (✓) indicates that the corresponding input is used or, in the target column, that forecasts are evaluated over the indicated target horizon. Blank cells indicate inputs that are not used.

Method	Station History t−23:t	Future NWP t+1:t+24	Coordinate Information	Target t+1:t+24
Raw NWP		✓		✓
Transformer/PatchTST/DLinear	✓	✓		✓
GWNet/MegaCRN	✓	✓	✓	✓
TF-STNet	✓	✓	✓	✓

**Table 5:** Five-seed test-set comparison with the best learned comparator by mean MAE for each task. Values are mean ± standard deviation over seeds 2026, 2025, 2024, 2023, and 2022. PCC is Pearson correlation; a negative reduction indicates that the proposed model has higher MAE.

Region	Variable	TF-STNet		Best Comparator			MAE Reduction
		MAE	PCC	Method	MAE	PCC	vs. Comparator
Shandong	Wind	0.676±0.001	0.700±0.000	MegaCRN	0.702±0.003	0.677±0.001	3.7%
	Pressure	0.353±0.144	0.998±0.002	Transformer	0.421±0.068	0.997±0.000	16.2%
	RHU	7.000±0.374	0.855±0.013	DLinear	7.865±1.054	0.801±0.055	11.0%
	Temperature	0.485±0.017	0.996±0.000	MegaCRN	0.430±0.082	0.997±0.001	−12.8%
Fujian	Wind	0.709±0.005	0.634±0.003	GWNet	0.739±0.007	0.602±0.009	4.1%
	Pressure	0.405±0.010	0.990±0.000	MegaCRN	0.552±0.159	0.980±0.011	26.6%
	RHU	5.003±0.044	0.893±0.001	GWNet	5.313±0.263	0.861±0.019	5.8%
	Temperature	0.992±0.016	0.960±0.001	GWNet	1.067±0.100	0.940±0.012	7.0%
Sichuan	Wind	0.640±0.003	0.524±0.003	Transformer	0.645±0.001	0.501±0.001	0.8%
	Pressure	1.082±0.077	0.954±0.005	MegaCRN	1.303±0.255	0.902±0.035	17.0%
	RHU	6.131±0.080	0.872±0.002	MegaCRN	6.222±0.067	0.856±0.006	1.5%
	Temperature	1.124±0.018	0.958±0.001	Transformer	1.279±0.081	0.945±0.006	12.1%
Hebei	Wind	0.606±0.005	0.688±0.001	GWNet	0.621±0.004	0.658±0.002	2.4%
	Pressure	0.467±0.026	0.997±0.000	GWNet	1.095±0.186	0.984±0.005	57.4%
	RHU	7.487±0.235	0.870±0.003	GWNet	7.690±0.017	0.849±0.004	2.6%
	Temperature	1.078±0.017	0.983±0.001	Transformer	1.147±0.247	0.969±0.010	6.0%

**Table 6:** Regional NMAE of the compared methods and the four-region mean. Lower is better.

Method	Shandong	Fujian	Sichuan	Hebei	Mean
NWP	1.000	1.000	1.000	1.000	1.000
Transformer	0.521	0.557	0.524	0.658	0.565
PatchTST	0.624	0.733	0.678	0.834	0.717
DLinear	0.507	0.551	0.543	0.733	0.584
GWNet	0.460	0.522	0.511	0.629	0.531
MegaCRN	0.455	0.537	0.502	0.732	0.558
TF-STNet	0.406	0.488	0.475	0.555	0.481

**Table 7:** Pressure MAE for simple MOS corrections and TF-STNet.

Region	Mean-Bias MOS	Linear MOS	TF-STNet	Reduction vs. Mean Bias	Reduction vs. Linear
Shandong	3.613	3.103	0.487	86.5%	84.3%
Fujian	1.804	1.382	0.405	77.5%	70.7%
Sichuan	1.943	1.488	1.082	44.3%	27.3%
Hebei	2.538	2.494	0.467	81.6%	81.3%

**Table 8:** Lead-time error summary for TF-STNet. Each cell gives mean MAE across leads, followed by the minimum-lead and maximum-lead errors (hours).

Region	Wind	Pressure	RHU	Temperature
Shandong	0.671 (15/6)	0.474 (14/8)	6.367 (22/8)	0.614 (4/24)
Fujian	0.707 (19/8)	0.411 (16/8)	5.088 (22/9)	0.917 (20/9)
Sichuan	0.639 (24/8)	0.730 (17/1)	6.116 (23/10)	1.130 (19/9)
Hebei	0.598 (20/7)	0.441 (13/24)	7.401 (1/11)	1.035 (18/8)

Numbers in parentheses are the lead with the minimum MAE/the lead with the maximum MAE.

**Table 9:** High-wind and rapid-change events. Improvements are relative MAE reductions; positive values favor TF-STNet.

Region	Event	Samples	NWP	MegaCRN	TF-STNet	vs. NWP	vs. Mega
Fujian	High wind	11,555	2.766	1.849	1.808	34.6%	2.2%
Fujian	Rapid change	12,511	1.881	1.461	1.447	23.1%	1.0%
Shandong	High wind	9920	2.026	1.749	1.613	20.4%	7.8%
Shandong	Rapid change	14,864	1.602	1.370	1.322	17.5%	3.5%
Hebei	High wind	9443	1.427	1.687	1.523	−6.7%	9.7%
Hebei	Rapid change	15,605	1.447	1.308	1.257	13.1%	3.9%
Sichuan	High wind	23,992	2.491	2.308	2.008	19.4%	13.0%
Sichuan	Rapid change	26,232	1.725	1.534	1.410	18.3%	8.1%

**Table 10:** Computational cost on an NVIDIA RTX 3080 Ti (batch size 32). Times are seconds per batch.

Region	Model	Train/Batch (s)	Inference/Batch (s)	Params (M)
Shandong	GWNet	0.1564	0.0462	0.4943
	MegaCRN	0.2750	0.0923	0.0296
	TF-STNet	0.1253	0.0482	0.8863
Fujian	GWNet	0.0959	0.0273	0.4923
	MegaCRN	0.2051	0.0767	0.0276
	TF-STNet	0.0961	0.0262	0.8365
Sichuan	GWNet	0.1737	0.1031	0.4957
	MegaCRN	0.2997	0.1590	0.0310
	TF-STNet	0.2116	0.1192	0.9413
Hebei	GWNet	0.1317	0.0622	0.4955
	MegaCRN	0.2456	0.1202	0.0309
	TF-STNet	0.1590	0.0664	0.9344

**Table 11:** Relative MAE of the ablation variants by region. Values are normalized to complete TF-STNet (1.000); values above 1 indicate degradation.

Variant	Shandong	Fujian	Sichuan	Hebei	Mean
w/o NWP	1.970	1.550	1.662	2.095	1.819
w/o Obs.	1.303	1.078	1.098	1.139	1.154
w/o Time	1.807	1.345	2.899	1.487	1.885
w/o Freq.	1.759	1.247	1.308	1.269	1.396
TF-STNet	1.000	1.000	1.000	1.000	1.000

**Table 12:** Variable-specific hyperparameter selections from one-factor-at-a-time validation sweeps. The selected value and corresponding validation MAE are reported in separate columns for each sweep.

Region	Variable	Neighborhood Sweep		Dimension Sweep	
		Best K	Validation MAE	Best D	Validation MAE
Shandong	Wind speed	5	0.671	128	0.671
	Pressure	10	0.324	64	0.318
	Relative humidity	2	6.255	256	6.410
	Temperature	9	0.468	256	0.477
Fujian	Wind speed	4	0.707	128	0.707
	Pressure	3	0.362	32	0.365
	Relative humidity	8	4.880	256	4.872
	Temperature	2	0.876	128	0.896
Sichuan	Wind speed	7	0.639	64	0.639
	Pressure	7	0.617	32	0.628
	Relative humidity	4	6.199	128	6.131
	Temperature	3	1.092	64	1.095
Hebei	Wind speed	6	0.598	256	0.598
	Pressure	7	0.405	64	0.379
	Relative humidity	6	7.192	128	7.415
	Temperature	2	1.043	64	1.046

## Data Availability

The data supporting the findings of this study were provided by the State Key Laboratory of Renewable Energy Grid-Integration, China Electric Power Research Institute. The data are not publicly available but may be obtained from the corresponding author upon reasonable request, subject to approval by the data provider. The complete experimental configuration is reported in Section 3.3. The model implementation, configuration files, and a wind-speed training and inference example are available at https://github.com/gyla1993/TS-STNet_source_code (accessed on 3 September 2026).

## Abbreviations

The following abbreviations are used in this manuscript:

NWP	Numerical weather prediction
KNN	*K*-nearest-neighbor operator
TF-STNet	Time–frequency dual-branch spatiotemporal network
GWNet	Graph WaveNet
MegaCRN	Meta-graph convolutional recurrent network
GCN	Graph convolutional network
MOS	Model output statistics
MAE	Mean absolute error
PCC	Pearson correlation coefficient
NMAE	Normalized mean absolute error
RHU	Relative humidity
TMP	Temperature
MLP	Multilayer perceptron
